# Antibiotic use in tonsillectomies: therapeutic or prophylactic? Required or excessive?

**DOI:** 10.1016/S1808-8694(15)31275-1

**Published:** 2015-10-20

**Authors:** Otávio Bejzman Piltcher, Fabiana Bortoncello Scarton

**Affiliations:** 1 Master degree in Medical Sciences; Faculdade de Ciências Médicas da Santa Casa de São Paulo; PhD in Medical Sciences; Ph.D. in Medicine, Faculdade de Ciências Médicas da Santa Casa de São Paulo and Pittsburgh University, US. Doctor hired by the ENT Service, Hospital de Clínicas of Porto Alegre.; 2 Internist, ENT Service, Hospital de Clínicas of Porto Alegre.

**Keywords:** antibiotics, tonsillectomies, prophylaxis, post-operative morbidity

## Abstract

Adenotonsillectomy is one of the most commonly performed surgeries in the pediatric and young adult populations. The postoperative morbidity of this surgical procedure is often significant, including odynophagia, dysphagia, fever, halitosis, loss of weight and reduced oral intake. After tonsillectomy, with or without adenoidectomy, the colonization of the open tonsillar fossae by the bacterial population of the oral cavity would cause an exacerbation of the local inflammatory response worsening postoperative pain. The hypothesis that a reduction of the bacterial population of an open surgical wound could minimize the local inflammation, stimulate the healing process and hasten recovery triggered a considerable number of studies addressing the relation between the use of perioperative antibiotics and postoperative morbidity of adenotonsillectomy. In spite of the fact that those studies claim to perform an assessment of the surgical prophylactic use of antibiotics, their outline is not in compliance with the worldwide-accepted principles of surgical antibiotic prophylaxis. By performing a critical review of the literature, the authors discuss the advantages and disadvantages of using antibiotics in tonsillectomies or adenotonsillectomies, as well as the most appropriate definition for its utilization.

## INTRODUCTION

Adenotonsillectomy is one of the most commonly performed surgeries among pediatric and young adults populations^1^. Although severe complications are rare, postoperative morbidity is often significant and symptoms include; odynophagia, dysphagia, otalgia, fever, halitosis, body weight loss and decreased oral intake.^1^, ^2^ Those symptoms may prolong convalescence and might occasionally require admittance to hospital for IV hydration and proper analgesia therapy.^1^

After tonsillectomy procedure performed with or without adenoidectomy the colonization of the open tonsillar fossa by oral bacterial flora may cause severe localized inflammatory reaction with subsequent pain exacerbation after the tonsillectomy.^2^ Therefore, it is reasonable to assume that reduction of bacterial population at the open surgical wound may decrease local inflammation, promote healing process and accelerate recovery.^3^

This hypothesis originated several studies that addressed the relation between perioperative use of antibiotics and postoperative morbidity of adenotonsillectomy. There are several well designed studies available in the literature (level A evidence), advocating the use of antibiotics to decrease postoperative morbidity of tonsillectomy (fever, halitosis, oral feed intake and return to normal routine).^1^, ^2^, ^3^, ^4^ Although these studies define themselves as designed to evaluate prophylactic surgical use of antibiotics in such surgeries, there were no follow up about internationally accepted surgical antibiotic prophylaxis, that is, up to two hours preoperatively until six hours postoperatively.^5^

The objective of this article is to discuss pros and cons of the use of antibiotics in tonsillectomy through a critical review of literature to properly define its use.

## LITERATURE REVIEW

One may state that there is unanimous consensus in the literature considering tonsillectomy with or without adenoidectomy as a surgical procedure with high postoperative morbidity.^1^, ^2^, ^3^, ^5^, ^6^ Adult patients commonly require up to two weeks to fully recover after tonsillectomy.^3^

Besides the trauma of the surgery itself, which includes all variables resulting from the technique applied (cold dissection/cold knife technique, electric/hot knife dissection, laser, coblation etc.), the key tissue reaction to the presence of microorganisms is inflammation.^7^ After tonsillectomy, with or without adenoidectomy, the colonization of the open tonsillar fossa by the oral bacterial flora may cause severe local inflammatory reaction exacerbating postoperative pain^1^. Pain and discomfort are symptoms of significant magnitude postoperatively, even after therapy with narcotic drugs.^3^

Oropharyngeal pain after tonsillectomy derives from the tonsillar fossa. The exposure of nervous terminals and the action of chemical inflammatory mediators such as lactic acid, leukotrienes and prostaglandins resulting in muscle spam are the cause of such pain. On the other hand, muscle spasm result in ischemia and a prolonged pain cycle.^4^ The pain-related issues resulting from such inflammatory healing process may include hospitalization for IV hydration and pain killer therapy.^1^, ^3^, ^6^

In 1956, Orzac tested the use of oral antibiotics therapy preoperatively and IM postoperatively to decrease morbidity and detected pain relief after tonsillectomy, lower number of hemorrhage events and of several types of infection.^8^ In 1986, Telian carried out the first clinical trial, a prospective, randomized, controlled study widely mentioned in the literature to test the effects of antibiotic drugs in tonsillectomy recovery in pediatric patients. He used Ampicillin, Sodium Salt or IV saline solution preoperatively followed by a seven-day course of amoxicillin or placebo preoperatively. The results showed significant improvement of the following criteria controlled for the group treated with antibiotic therapy against the placebo group: fever, return to normal diet, reduced intervals of pain, less odor of the oral cavity, and faster return to normal routine.^2^ The return to normal activities, whether it was school or work, was used as indicators of recovery and well being.^3^ In another prospective, randomized, double-blind, placebo-controlled study, which was conducted in adult patients this time, Grandis et al. also found significant differences related to faster return to normal diet, less odor intensity of the oral cavity and faster return to normal routine. As to pain, however, only some decreasing trend was reported. This study had 101 patients (age 12 or +) that have undergone tonsillectomy, with or without adenoidectomy. Patients received the first dose of IV antibiotic therapy after tonsil removal (ticarcillin + clavulanic acid). The control group received sterile saline infusion. Two other doses of IV antibiotic therapy were given within 6-hour interval. Patients in the antibiotic therapy group received amoxicillin with clavulanic acid (250 mg every 8 hours for seven days). The lower morbidity level in the group treated with antibiotics was clinically significant and the relative theory of its use was biologically plausible, or in other words, perioperative eradication of bacteria in open wound would reduce chances of infection, expedite the healing process and accelerate recovery. The authors also emphasized that although a significant difference was not detected regarding side effects in the group treated with antibiotic therapy, low incidence of adverse effects in this relatively small sized sample reduced the likelihood of detecting a rare or more severe side effect such as hemolytic anemia or anaphylaxis.^3^

In a more recent not randomized and not double-blind clinical trial, Colreavy et al. compared two groups of children: Group A, treated with amoxicillin-clavulanate for seven days after tonsillectomy, and Group B, the control group. In this study, pediatric patients treated with antibiotic therapy postoperatively presented considerably lower morbidity if compared against the control group in some criteria such as reduced requirement for pain killer therapy (p=0.038), earlier return to normal diet (p=0.0072) and better pain scores (p=0.0006).^4^

In 1999 a prospective, randomized, double blind, placebo-controlled study including 36 (thirty-six) adult patients that had undergone tonsillectomy sub-divided the sample into four groups. The first group received only placebo, the second group received ampicillin transoperatively and amoxicillin-clavulanate or ticarcillin-clavulanate respectively preoperatively and 8 hours postoperatively. The results showed that topical use perioperatively of ticarcillin/clavulanate or amoxicillin/clavulanate or clindamicin resulted in reduction of oropharyngeal pain and odor in oral cavity if compared against control group and against ampicillin followed by amoxicillin both given through systemic route.^1^

Some studies in the literature suggest that infection of the tonsillar fossa may contribute or even be the cause of secondary hemorrhage. The estimates in the literature about the incidence of postoperative hemorrhage vary from 0-20%. Regarding the role of transoperative use of antibiotics in the prevention of such complication, there is a study published in 1986, proposing that one of the reasons for low bleeding rate could be antibiotic therapy after the surgery. In this study 38 pediatric patients submitted to tonsillectomy totalizing 1,445 children had postoperative bleeding, amounting to percentage of 2.62 .^10^

The only reason established for the use of antibiotic drugs on a prophylactic basis for patients that will undergo tonsillectomy is to prevent endocarditis and septicemia. The relation between postoperative bacteremia and development of septicemia with consequent endocarditis in patients with heart valve disease has been established by several authors^11^, ^12^ Although heart valve disease is currently less common, surgeons are more aware about the need of prophylactic use of antibiotics in patients with implants, such as Splintz-Holter valves and devices of orthopedic fixation in situ. Transient bacteremia under such circumstances could lead to the development of severe infections of this sites.^11^

Another favorable aspect that emphasizes the use of antibiotics in tonsillectomy, since it has the purpose to reduce the incidence of postoperative infection and subsequently all events resulting from it, is that prophylactic antibiotic therapy may reduce global costs preventing expenses related to infection and reducing hospital stay.^13^

With respect to the type of antibiotic to be used, changes in microbiological profile of the tonsillar tissue over the last ten years have been affecting this choice.^4^ In the study of Telian et al., ampicillin and amoxicillin were used aiming at the coverage of *Streptococcus Pyogenes*, which was the pathogen responsible for 90% of chronic tonsillitis cases. But, over the last decade, there have been increased evidence that *Haemophilus influenzae* and *Staphylococcus aureus* may play a key role in such infections.^4^ DeDio et al., in his review of the microbiology of palatine tonsils and adenoids, issued in 1988, identified *Haemophilus influenzae* and *Staphylococcus aureus* as the most common pathogens in these tissues. Both microorganisms produce ß-lactamases and are characterized by multi-resistant to antibiotics. *Haemophilus sp* was identified in 54%, and *S. Aureus sp in* 46% of his patients. Only 8% of all isolated *S. aureus* showed sensitivity to penicillin G or amoxicillin. Still in this study, 17 to 20% of the species of *Haemophilus* produced ß-lactamase. ^14^ Based on these facts, Mevio et al. concluded that amoxicillin-clavulanate would be the most effective antimicrobial drug to eradicate such microorganisms, and his opinion is also shared by Grandis et al and others.^3^, ^4^ Additionally, this type of coverage would act in anaerobic organisms that are also present in this site.^4^

On the other hand, the idea that the use of a broad-spectrum antibiotic drug would offer better results was not confirmed in the study by Jones et al. The authors compared the efficacy of Cefaclor against amoxicillin in the recovery of children after tonsillectomy. Results showed that there were no significant differences between the two groups regarding intensity or duration of symptoms postoperatively, and the same happened in terms of complications.^15^

There is only one study that reports unfavorable results related to postoperative use of antibiotics in recovery of pediatric patients that have undergone tonsillectomy. In this study, 54 children received antibiotic therapy postoperatively against 41 children that did not receive anything. There was not significant reduction in any of the measures of morbidity in patients under antibiotic therapy. In fact, the amount of painkillers and the incidence of otalgia, symptoms of irritability on the sixth and seventh day, and secondary hemorrhage were higher in the group under antibiotic therapy.^16^

### Prophylaxis with antibiotic therapy for Surgery

The prophylaxis using antibiotic therapy in surgeries is related to the use of antibiotic in patients without any evidence of established infectious process with the objective to prevent systemic infection or operative wound infection that might occur.^17^ It is obtained by the reduction of the number of viable bacteria below the critical level in the wound to promote infectious process, in other words, 10 million per gram of tissue, except if there is presence of a foreign body (e.g. prosthesis).^7^, ^16^

The prophylactic use of antimicrobial drugs in surgery is indicated in surgical interventions with high probability of infection of the surgical wound, such as clean-contaminated surgeries or potentially contaminated surgeries, for surgeries in which septic complications represent total loss, such as surgical procedures to implant prosthesis and in immunedepressed patients^17^, ^18^ Elevated infection rates without antibiotic therapy and their reduction after its use, in clinical, controlled studies justify prophylaxis under such circumstances.^13^ In the case of contaminated and infected surgeries the indication of antibiotic therapy is made for curative and not prophylactic purpose in compliance with standards already established for conventional antibiotic therapy.^17^

Both the usual flora found during surgery and the organisms responsible for the postoperative infection affect the choice of prophylactic antibiotic drug to be used, but the coverage is oriented, primarily to those organisms that cause postoperative infection. There is some controversy in the literature about the drug to be used, considering in general antimicrobial drug cefazolin as first choice therapy (1g IV) in the preoperative period.^19^

Based on clinical trials it was concluded that to be fully effective the antibiotic must be present in proper concentrations at the surgical site and as early as possible in the decisive interval (around 3 hours after the beginning of the surgery) and during the interval in which the wound will remain open.^20^, ^21^

With respect to antimicrobial prophylaxis, the shorter effective course should be used. In most of the circumstances, it consists in antibiotic coverage just while the patient is in the surgery room. The most severe mistakes regarding surgical antibiotic prophylaxis are inappropriate use of broad-spectrum therapeutic agents and excessive administration for long time period.^5^

## DISCUSSION

The search for therapies able to mitigate high morbidity of tonsillectomies is totally understandable and desirable. The use of painkillers does not totally control pain and its consequences in the postoperative setting.

The hypothesis that the inflammatory process of traumatized tonsillar fossa is exacerbated and prolonged with proven presence of bacteria at this site leads several authors to test the use of antibiotics under this situation, known as prophylactic use.

The way these drugs were tested (trans-operative with seven day follow up) did not allow us to define it as prophylactic according to internationally defined standards and proven surgical prophylaxis. The use of antibiotics to reduce colonization of tonsillar fossa with the purpose to decrease symptoms resulting from healing inflammatory process, regardless of their efficacy, should be denominated therapeutic, even in the absence of an existing infectious process. Therefore, it could be possible to use the term prophylactic in the sense of prevention, at least partially, of severe post-tonsillectomy morbidity, especially regarding postoperative pain. The articles available, except for one, report a significant difference in favor of trans and operative use of antibiotics against control group, with decreased morbidity that is typical in such procedure. There is only one study proving the efficacy of antibiotic therapy for topical use and in the short run. This possibility requires further investigation.

Another questionable aspect relates to the criteria applied for the choice of antibiotics. The authors have been justifying the choice of broad-spectrum drugs and effect against b-lactamase based on the knowledge of bacterial flora present on surface and inner tonsillar tissue. Since tonsils are removed in the procedure and the oropharyngeal flora of the tonsillar fossa remains, there is a possibility that bacteria responsible for the inflammatory response will not be detected in the tonsils.

There are no NNT (number-needed-to-treat) calculations available. This type of statistical approach could be useful to weight risks and benefits related to the use of antibiotics in tonsillectomies as tested in the literature in more practical terms. There is a possibility that the type of surgical technique may have an impact in this outcome, in the US where those studies were conducted most of the centers use thermal cautery, known to cause more postoperative pain. The individual and community risks related to increased bacterial resistance, potential side effects of antibiotic therapy (diarrhea, allergic reactions etc) and treatment costs should be carefully taken into account against the likely benefit of lower pain interval, faster return to normal feeding and fewer repeated interventions.

Today, the management approach of the authors is to restrain the use of antibiotics as therapy to provide better postoperative care in adult patients that are likely to have worse outcomes. Possibly, the pediatric patient population present lower morbidity level due to the easiness to remove tissues, and to the type of technique generally applied (cold dissection). The prophylactic use itself will keep on being used in those cases with established indication (prosthesis, heart valve disease etc). Two research protocols were established after this review, one to detect tonsillar fossa flora and the other to evaluate the use of antibiotic therapy exclusively related to prophylaxis, both topical and systemic, in the outcome of this type of surgical procedure.
